# *Streptococcus pyogenes *carriage acquisition, persistence and transmission dynamics within households in The Gambia (SpyCATS): protocol for a longitudinal household cohort study

**DOI:** 10.12688/wellcomeopenres.18716.2

**Published:** 2023-10-30

**Authors:** Edwin P. Armitage, Alex J. Keeley, Gabrielle de Crombrugghe, Elina Senghore, Fatoumatta E. Camara, Musukoi Jammeh, Amat Bittaye, Haddy Ceesay, Isatou Ceesay, Bunja Samateh, Muhammed Manneh, Abdul Karim Sesay, Beate Kampmann, Adam Kucharski, Thushan I. de Silva, Michael Marks, MRCG StrepA Study Group

**Affiliations:** 1 Vaccines and Immunity Theme, Medical Research Council Unit The Gambia at the London School of Hygiene & Tropical Medicine, Banjul, The Gambia; 2 Molecular Bacteriology Laboratory, Faculty of Medicine, Free University of Brussels, Brussels, Belgium; 3 Genomics Strategic Core Platform, Medical Research Council Unit The Gambia at the London School of Hygiene & Tropical Medicine, Banjul, The Gambia; 4 Centre for Mathematical Modelling of Infectious Diseases, London School of Hygiene and Tropical Medicine, London, WC1E 7HT, UK; 5 The Florey Institute and Department of Infection, Immunity and Cardiovascular Disease, Medical School, University of Sheffield, Sheffield, S10 2TN, UK; 6 Division of Infection and Immunity, University College London, London, WC1E 6BT, UK; 7 Department of Clinical Research, Faculty of Infectious and Tropical Diseases, London School of Hygiene & Tropical Medicine, London, WC1E 7HT, UK; 8 Hospital for Tropical Diseases, University College London Hospital, London, NW1 2BU, UK

**Keywords:** Streptococcus pyogenes, asymptomatic carriage, pharyngitis, pyoderma, longitudinal cohort study, transmission modelling, The Gambia, rheumatic heart disease

## Abstract

**Background:**

*Streptococcus pyogenes* (StrepA) causes a significant burden of disease globally from superficial infections to invasive disease. It is responsible for over 500,000 deaths each year, predominantly in low- and middle-income countries (LMIC). Superficial StrepA infections of the skin and pharynx can lead to rheumatic heart disease, the largest cause of StrepA-related deaths in LMIC. StrepA can also asymptomatically colonise normal skin and the pharynx (carriage), potentially increasing infection risk. *Streptococcus dysgalactiae subsp. equisimilis* (SDSE) carriage is also common in LMIC and may interact with StrepA. This study aims to investigate StrepA and SDSE carriage and infection epidemiology, transmission dynamics and naturally acquired immunity within households in The Gambia.

**Methods:**

A longitudinal household observational cohort study will be conducted over one year. 45 households will be recruited from the urban area of Sukuta, The Gambia, resulting in approximately 450 participants. Households will be visited monthly, and available participants will undergo oropharyngeal and normal skin swabbing. Incident cases of pharyngitis and pyoderma will be captured via active case reporting, with swabs taken from disease sites. Swabs will be cultured for the presence of group A, C and G beta-haemolytic streptococci. Isolates will undergo whole genome sequencing. At each visit, clinical, socio-demographic and social mixing data will be collected. Blood serum will be collected at baseline and final visit. Oral fluid and dried blood spot samples will be collected at each visit. Mucosal and serum anti-StrepA antibody responses will be measured.

**Outcome:**

This study will report StrepA and SDSE clinical epidemiology, risk factors, transmission dynamics, and serological responses to carriage and infection. Detailed social mixing behaviour will be combined with phylogenetic relatedness to model the extent of transmission occurring withing and between households. The study will provide data to help meet global strategic StrepA research goals.

## Introduction

*Streptococcus pyogenes* (Group A *Streptococcus*, StrepA) is a beta-haemolytic Gram positive bacterium that is a major cause of infectious disease burden globally, responsible for over 500,000 annual deaths ^1– 4^. It causes a wide spectrum of disease from superficial skin and pharynx infections to invasive disease, in addition to the immunological sequelae of acute rheumatic fever, rheumatic heart disease (RHD) and acute post-streptococcal glomerulonephritis ^3, 5^. Each year an estimated 1.8 million invasive StrepA, 111 million pyoderma and 616 million pharyngitis cases occur globally ^2^. Most clinical StrepA infections are thought to occur in low- and middle-income countries (LMIC), though data from such countries is lacking ^1, 2, 6, 7^. Moreover, RHD, the most serious immunological consequence of StrepA infection, causes over 300,000 deaths each year, predominantly in LMIC ^3^, where diagnosis and surveillance is poor ^3, 8^.

Despite the significant burden of StrepA disease and its immunological sequelae, there has been little focus on StrepA carriage and transmission in LMIC ^8, 9^. Furthermore, the understanding of the natural history of StrepA carriage, transmission and infection is limited ^9, 10^. A better understanding of carriage incidence, prevalence, persistence (duration), seasonal variation, transmission and the associated risk factors within high-disease burden settings in LMIC is crucial to design and implement interventions targeting StrepA in such countries.

The epidemiology of superficial StrepA infections in The Gambia is poorly understood. In 2018 ^11^, a cross-sectional study in 1441 Gambian children under five years old found a high prevalence of bacterial pyoderma (17.4%), scabies infestation (15.9%), and of StrepA culture-positive pyoderma (8.8%). There was also a significant increase in pyoderma detected during the rainy season compared to before (8.9% vs. 23.1%, adjusted prevalence ratio 2.42, CI 1.39-4.23).

Whole genome sequencing (WGS) has transformed our ability to understand StrepA epidemiology, giving significantly better resolution than *emm* typing to determine linkage between strains. This has been used to gain valuable insights into transmission dynamics and to inform outbreak investigation in HICs ^12– 16^. In low-income settings where the molecular epidemiology of StrepA is notably different, combining WGS data, clinical and behavioural data with mathematical models can provide new insights into transmission dynamics and potential intervention strategies ^17, 18^.

### Rationale

In 2018, the World Health Assembly stated that RHD and StrepA research should be a global priority ^19^. The WHO then published a Group A *Streptococcus* Vaccine Development Technology Roadmap highlighting key strategic areas for research including to improve global estimates of disease burden and epidemiology of StrepA infections, and to further describe the spectrum of natural disease history and immunity in longitudinal studies ^9, 10^. Our limited understanding of StrepA transmission dynamics and immunity is mostly derived from studies in high-income countries (HIC) ^20– 22^. However, in LMIC such as The Gambia, a higher prevalence and incidence of StrepA carriage and a wider diversity of the circulating *emm* types may underlie the higher burden of StrepA-related clinical infections and immune sequelae seen ^23– 25^.

Very few longitudinal studies of StrepA exist ^20, 26, 27^, and high-quality longitudinal data from a high-prevalence country in sub-Saharan Africa combining classical epidemiology with detailed social mixing behaviour and next generation WGS techniques to model disease transmission will be highly informative in growing our understanding of StrepA epidemiology and meeting global strategic StrepA research goals on the road towards a StrepA vaccine.

### Study objectives

Primary:

1. To determine the prevalence, incidence, duration and transmission dynamics of asymptomatic StrepA carriage and clinical StrepA infections within households.
2. To establish risk factors for pharyngeal and skin clinical StrepA infection, including detailed characterisation of the relationship with individual and household asymptomatic carriage, *emm* type and seasonality.
3. To develop a mathematical model of household StrepA transmission using clinical, behavioural and phylogenetic relatedness data to calibrate it, to allow for estimation of the relative contributions of between and within household transmission.

Secondary:

1. To determine risk factors for asymptomatic StrepA pharyngeal and skin carriage.
2. To describe the role of asymptomatic StrepA skin and pharyngeal carriage in StrepA transmission and infection.
3. To describe seasonal variation in StrepA carriage and clinical infection throughout the year.
4. To describe StrepA *emm* type diversity.
5. To investigate the extent of StrepA tissue tropism of *emm* types identified.
6. To determine the prevalence and incidence of *Streptococcus dysgalactiae subspecies equisimilis* (groups C and G streptococcus; SDSE) carriage and clinical infection.
7. To describe the prevalence, incidence and transmission dynamics of *Staphylococcus aureus* skin carriage and infection within households.
8. To describe variations in bacterial density by site, season and clinical characteristics using quantitative PCR.
9. To identify non-human sources of StrepA within households and the presence of airborne StrepA indoors using settle plates.
10. To describe the antimicrobial sensitivity of StrepA isolates identified.
11. To describe age-stratified anti-StrepA antibody titres.
12. To explore StrepA-specific serological and mucosal immune activity in response to colonisation and disease.
13. To investigate the relationship between anti-StrepA antibody titres and risk of incident colonisation and infection to explore serological correlates of protection.

## Protocol

### Study setting

The Gambia is a small country in West Africa with a population of approximately two million people. It was ranked 174th by the United Nations Human Development Index in 2021, making it one of the least developed countries in the world. It is a predominantly Muslim country, comprising several tribal groups, the largest being Mandinka, Wolof, Fula and Jola.

Sukuta is an urban area, part of the coastal region’s sprawling conurbation, where most of the population live. It is a majority Mandinka area, with a population of 47,048, and an average household size of 8.1 in the census in 2013.

The climate is sub-tropical with a long dry season from November to May, and a short rainy season between June and October each year.

### Study design

SpyCATS is a prospective, longitudinal (open) cohort study within households in Sukuta, The Gambia. Households will be recruited, and all household members present at the time of the visit will be asked to participate. Households will be followed for 12 months, with monthly visits, and more frequently for some subgroups of participants (described below).

A sample size target of 450 participants was determined (see below). With an average household size of approximately 10 people in Sukuta (based on our previous research and experience in the area given the census data is substantially out of date), 45 households will be recruited, and every available consenting household member included as an individual participant.

### Selection of participants

The study will enrol participants as individuals within households. Households will be identified using a process of GPS random selection. No complete sampling frame of households exists for Sukuta, however geographic information system data exist from the 2013 census of The Gambia. These data will be utilised to obtain a random set of GPS sampling locations stratified by population density. A list of GPS coordinates for the locations will be identified and for each location and the nearest household will be approached for participation. Each location on the list will be approached in order until the desired sample size is reached. Households will only be enrolled if over 50% of household members consent to participate in the study.

For the purposes of enrolment in the study, a household will be defined according to The Gambia Demographic and Health Survey 2013 definition: “a household [is] defined as a person or a group of related or unrelated persons who live together in the same dwelling unit(s) or in connected premises, who acknowledge one adult member as the head of the household, and who have common arrangements for cooking and eating.”

### Inclusion criteria

Households must:

- Be within the boundary of Sukuta as determined by the 2013 census
- Have at least 3 members including at least one child under age 18

Individuals must:

- Provide signed (or thumb-printed) informed consent for study participation (obtained from a parent or guardian for children under the age of 18)
- Be willing and have capacity to participate and comply with the study protocol as judged by a member of the study team
- Be resident in the household, with no plans to move outside of the household during the period of study participation

### Exclusion criteria

Households:

- Less than 50% of individuals living in the household, as defined by the The Gambia Demographic and Health Survey 2013 definition, provide consent to participate

Individuals:

- Consent not provided
- Has any condition or any other reason that may lead to difficulty or discomfort in obtaining all the necessary samples
- Is judged by the study team member to be unable or unlikely to participate and comply with the study protocol for the entire study period. Examples could include individuals with severe mental health conditions, communication barriers that cannot be overcome, or frequent absences from the household.

NB. There are no restrictions on enrolment of pregnant women or newly born babies.

### Field activities

**Figure 1.**
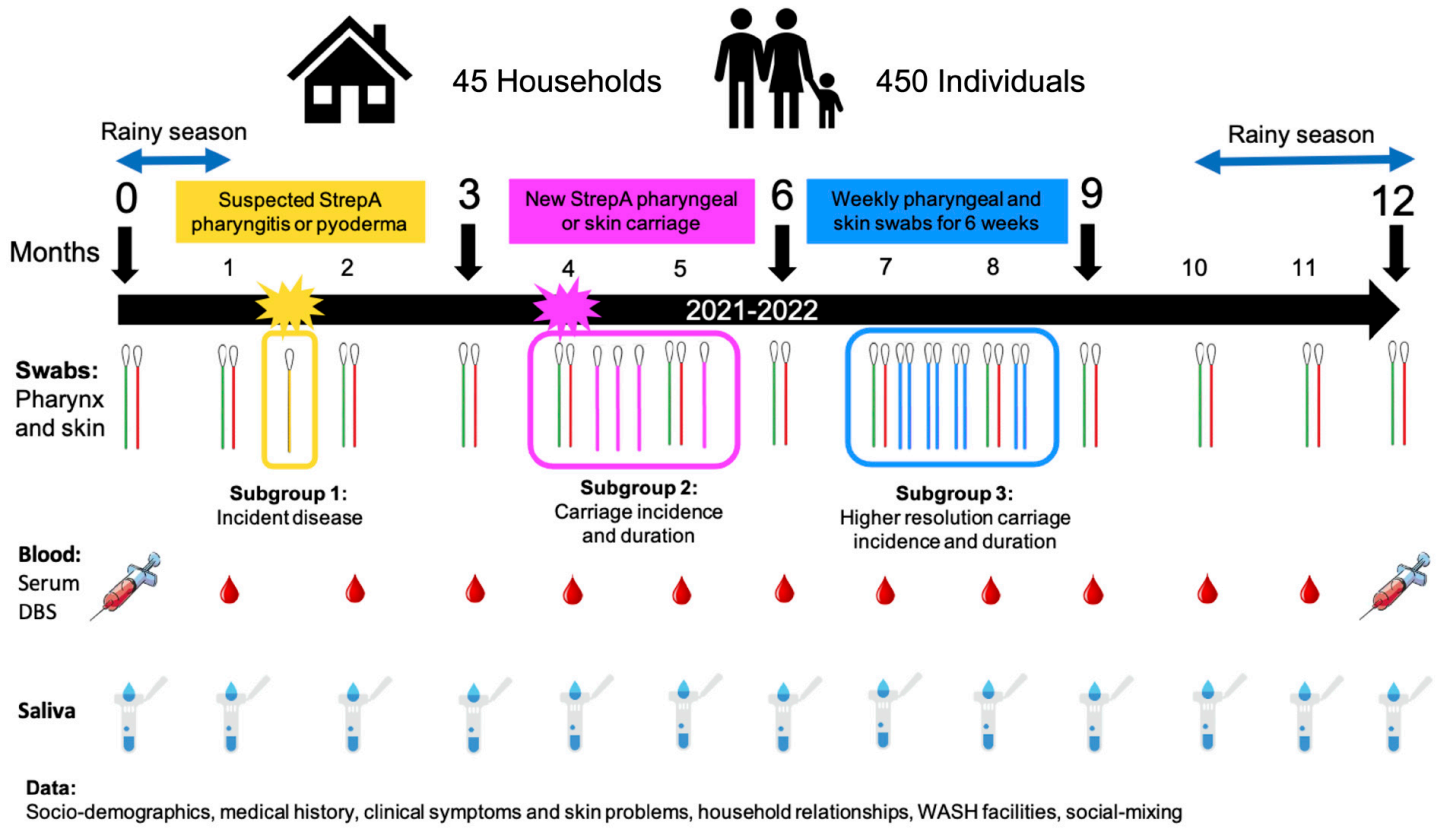
SpyCATS study diagram.

***Overview.*** Households will be enrolled for 12 months covering both the dry and rainy seasons, with enrolment having commenced in July 2021. Every household will undergo an enrolment visit (MV0), then monthly visits (MV1, MV2, MV3 etc., up to MV12) unless practical constraints arise (see Figure 1). At each visit, the household size will be determined by the number of individuals who slept in the household the previous night, and those household members present will be asked to participate. Household members not available to be seen will be still allocated an ID number, in order to capture relevant information regarding their social mixing with other household members, and if they are present at later visits, they will be asked to consent and enrol. Participants whose baseline (enrolment) visit occurs after MV0 will be asked why they were not available previously. Reasons for missed visits and late enrolment will be captured.

At each visit an oropharyngeal swab (OPS), normal skin swab (NSS), oral fluid sample (OF) and dried blood spot (DBS) will be taken from all enrolled individuals present and data collected on socio-demographics, social mixing behaviour and clinical examination findings. In addition, a blood sample for serum (BS) will be taken at the beginning and end of the study for detailed functional immune responses. Wound swabs (WS) will also be taken from any pyoderma lesions and swabs taken from non-infected skin overlying scabies lesions (scabies skin swab, SSS). Additionally, environmental swabs (ES) will be collected from common touch points in the household and settle plates (SP) placed inside households. Throughout the study, enrolled participants reporting symptoms potentially consistent with StrepA infection will have an unscheduled visit including a physical examination, an appropriate swab (OPS or WS), OF and DBS. Swabs will be transported the same day to the microbiology laboratory and plated for culture. The culture will identify the presence of catalase-negative, beta-haemolytic streptococci, and determine the group by latex agglutination testing. Antibiotic resistance will be determined, and any isolates will be stored in glycerol for later use. *Staphylococcus aureus* isolates cultured from NSS and WS will also be documented and stored for later characterisation. DBS and OF samples will be used for serological and mucosal immunology objectives. StrepA and SDSE isolates identified will undergo whole genome sequencing (WGS).

***Enrolment visit.*** At the enrolment visit (MV0), each individual will undergo a baseline survey including participant’s sociodemographic data, medical history, social mixing and behavioural factors. A physical examination will be carried out including a full body examination (taking care to maintain privacy) looking for any evidence of skin infections, and OPS and NSS will be collected. Additionally, an OF sample will be obtained, and venepuncture performed for BS and DBS. Any participant who is exhibiting signs and symptoms of pharyngitis (sore throat and pharyngeal inflammation) will be further examined and managed as they would be at an unscheduled visit for possible StrepA infection (see below). Any participant who is identified as having evidence of pyoderma (bacterial skin infection involving pus or crusts), will have a WS taken, and will be provided antibiotic treatment according to local guidelines as appropriate. Any additional abnormal finding requiring further investigation or treatment will be managed according to local practice or an appropriate referral made according to the nature of the finding.

Alongside the questionnaires on individuals, data will be collected on households such as household setup. Two ES will be taken from common touch points such as door handles and soft furnishings and a SP will be placed indoors to investigate airborne transmission.

***Monthly visits (MV1, MV2, MV3 etc. up to MV12).*** Participants will be enrolled for 12 months undergoing an enrolment visit (MV0), then monthly visits (MV1, MV2, MV3 etc. up to MV12), though due to the open cohort design new participants can enrol at visits later than MV0 (this includes babies born during the study period if their parent/guardian consents).

At each monthly visit following enrolment, the study team will collect further survey data from each individual household member, collect an OPS, NSS, OF and DBS. Survey data collected will include socio-demographic information, social mixing, behavioural factors and clinical examination findings. Participants with any evidence of pyoderma will have a WS taken and will be offered antibiotic treatment; any other abnormal finding requiring further investigation or treatment will be managed according to WHO guidelines or best local practice or an appropriate referral made according to the nature of the finding. Additionally, any use of antibiotics or other medication, or any attendance at a healthcare setting since the previous visit will be recorded.

***Unscheduled visits for possible StrepA infections.*** All enrolled households will be provided with an on-call number to call at any time when an enrolled household member is experiencing symptoms that could be compatible with a StrepA infection or any other acute illness. A study nurse will then complete a rapid assessment over the phone and arrange for an unscheduled visit as appropriate.

A study nurse will collect data on the history of the complaint, any medication taken and other relevant information. They will perform a physical examination, including vital signs under the supervision of a study clinician. If the symptoms are consistent with possible StrepA infection (pharyngitis or pyoderma), then a study swab will be taken (OPS or WS) for culture alongside an OF and DBS sample. For cases of pharyngitis and pyoderma, treatment will be provided empirically according to locally devised guidelines based on WHO and local guidelines and medication availability (see Table 1).

**Table 1.** SpyCATS empirical treatment guidelines for potential StrepA infections.

Diagnosis	Signs and symptoms	First-line treatment	Alternative treatment
Pyoderma	Purulent or crusted skin lesion	Cloxacillin (weight-based dose for children under 12 years; over 12 years 500mg qds 5–7 days)	Azithromycin (12 mg/kg up to max 500mg od 5 days)
Pharyngitis	Sore throat, pharyngeal/ tonsillar erythema	Penicillin V (weight-based dose for children under 12 years; over 12 years 500mg qds 5–7 days)	Azithromycin (12 mg/kg up to max 500mg od 5 days)

Participants presenting with symptoms of systemic infection or possible invasive disease will be urgently referred to the MRCG clinical services department for management. Participants presenting with other minor acute medical complaints will be provided appropriate treatment by the study team. All other medical presentations will be referred to the appropriate local healthcare facility.

### Sub-studies

***Intensified incident surveillance cohort.*** A subgroup of 16 randomly selected households will undergo intensified swabbing. This subgroup will be used to assess incidence and duration of StrepA and SDSE carriage and disease with greater resolution than the main cohort. During the second half of the study, the 16 households will undergo blocks of 6 weeks of intensified swabbing in batches of 4 households in turn, until all 16 have had a 6-week block of weekly swabbing. The households will be visited weekly for each of the 6 weeks of the intensive swabbing period. All household members present will undergo swabbing (OPS and NSS) in addition to more detailed social mixing behaviour data being collected. These data will be used in combination with WGS data from any StrepA isolated to inform the household transmission model. 

***Estimating duration of StrepA carriage.*** Following MV0, any participant who becomes an asymptomatic StrepA (or SDSE) carrier (i.e. was negative at baseline or the previous visit, and then becomes positive at a monthly visit without symptoms) will have weekly swabs taken from the same site that was positive (OPS or NSS), until two negative swabs have been received in a row. 

***Nested cross-sectional study of personal hygiene behaviour.*** At a MV11 or MV12, participants will be requested to undergo an additional survey on their personal hygiene behaviours during the last week including laundry, handwashing, bathing and soap and disinfectant use. Attitudes towards wound care and usual practice of participants in response to wounds will be captured.

At the same visit, additional environmental swabs (ES) will be collected from the household including four commonly touched locations within the household and a sample of water from the main household greywater source.

These data, combined with individuals’ carriage and infection data from the wider study will be used to assess the relationship between individual- and household-level hygiene behaviours and StrepA, SDSE and *S. aureus* carriage, infection and non-human source presence within households in this setting.

### Clinical and field evaluations

***Socio-demographics and household set-up.*** At the enrolment and later visits where necessary, a questionnaire will be asked of each individual participant in relation to their socio-demographic information including their date of birth, sex, tribal group, educational level and occupation. Any relevant medical information that is identifiable from ante-natal cards (ANC), or infant welfare cards (IWC) (especially for younger children) will be recorded such as birthweight, previous medical diagnoses and allergies.

For each household, data will be collected relating to the household set-up including the number of buildings, family relationships, number of rooms, accessibility for non-household members, sleeping arrangements, mosquito net use, water access, sanitation and hygiene (WASH) facilities and proximity to community meeting points.

At subsequent monthly visits, individuals will be asked to update some of their sociodemographic details such as occupation, work place, school name and attendance and any other factors that may change throughout the year, and to complete any missing data. Similarly, alterations to household set-up will also be collected.

***Social mixing behaviour.*** For all participants at their enrolment and monthly visits, and in more detail for the intensified incidence cohort at each weekly visit, data will be collected on individuals’ social mixing behaviour. Participants will be asked information about which other household members they had close contact with the previous day, and who they shared a food bowl, room and/or a bed with. They will be asked about their non-household social contacts from the previous day, including each contacts age, relationship to them, the location of meeting, whether there was physical contact and the duration. For the more detailed extended social mixing behaviour collected in the intensified swabbing cohort, the above data will also be collected for household social contacts.

***Medical and drug history.*** At enrolment, a focused past medical history will be taken from participants including any regular medication taken, previous diagnoses and previous history of skin or throat infections and acute rheumatic fever specifically. At enrolment and at each subsequent visit a brief history of recent medication (particularly antibiotics) and current clinical symptoms will be taken, including details of any recent healthcare setting attendance including traditional healers.

At unscheduled visits, a clinical history of the presenting complaint, medication usage and healthcare attendance will be taken to capture information related to any potential StrepA infections, but also to inform immediate and subsequent medical management of other complaints.

***Clinical examination and vital signs.*** At the enrolment visit, all participants will undergo a physical examination including vital signs to provide a baseline. Vital signs collected will include axillary temperature, pulse rate and respiratory rate. Adults (over 18 years) will also have blood pressure recorded.

Participants will then undergo a physical examination which will include an examination of the pharynx and associated lymph nodes, and a full body examination of the participant’s skin, to identify any pyoderma lesions, and other relevant skin conditions (particularly scabies and fungal infections). Care will be taken to perform the full body examination with appropriate privacy and verbal consent obtained at the time. Participants’ genitals will only be examined if they specifically report (or the parent reports, in the case of children) the presence of a lesion and verbally consent for the study nurse examine them.

At each subsequent visit participants will undergo the physical examination as described including throat and skin but will not have vitals recorded unless they are reporting any symptoms. If they have any medical complaint, a clinical history will be taken and fuller clinical examination of the presenting complaint will be done, in addition to recording vital signs. At unscheduled visits participants will also have their vitals recorded in the same way, and a clinical history and focused clinical examination will be done.

### Clinical samples

At each visit, participants will have clinical samples collected according to the sampling schedule outlined in Table 2.

**Table 2.** Visit data and sampling schedule for the various cohorts. *only at MV0 and MV12.

Visit timing	Visit window	Data and samples	Main cohort	Intensive incident cohort (16 households)	Duration cohort (new carriers)
Month 0 enrolment visit (MV0)	-	Eligibility	X	-	-
		Socio-demographics	X	-	-
		Social mixing behaviour	X	-	-
		Household setup and WASH	X	-	-
		Previous and recent medical history	X	-	-
		Pharyngeal and skin examination including vitals	X	-	-
		Oropharyngeal swab	X	-	-
		Normal skin swab	X	-	-
		Blood serum ^*^	X	-	-
		Dried blood spot	X	-	-
		Oral fluid	X	-	-
		Environmental swabs (x2)	X	-	-
		Settle plate	X	-	-
Weekly visits for duration and intensive incident surveillance	+/- 7 days	Recent medical history	-	X	X
		Extended social mixing behaviour	-	X	-
		Pharyngeal and skin examination	-	X	X
		Oropharyngeal swab	-	X	(X) *if previously* *positive*
		Normal skin swab	-	X	(X) *if previously* *positive*
Monthly visits (MV1, MV2, MV3 etc. up to MV12)	+/- 14 days	Update socio-demographics, household setup and WASH	X	X	-
		Recent medical history	X	X	-
		Social mixing behaviour	X	-	-
		Extended social mixing behaviour	-	X	-
		Pharyngeal and skin examination	X	X	-
		Oropharyngeal swab	X	X	-
		Normal skin swab	X	X	-
		Blood serum ^*^	X	X	-
		Dried blood spot	X	X	-
		Oral fluid	X	X	-
		Environmental swabs (x2)	X	X	-
		Settle plate	X	X	-
Unscheduled visits (may occur at scheduled visits if symptoms present)	-	Clinical history and examination	X	X	X
		Wound or oropharyngeal swab	(X) *if applicable*	(X) *if applicable*	(X) *if applicable*
		Oral fluid	(X) *if applicable*	(X) *if applicable*	(X) *if applicable*
		Dried blood spot	(X) *if applicable*	(X) *if applicable*	(X) *if applicable*
		Scabies skin swab	(X) *if applicable*	(X) *if applicable*	(X) *if applicable*
Personal hygiene visit (done at another monthly visit)	-	Personal and household hygiene behaviour	X		
		Extended environmental swabs (x5)	X		

***Oropharyngeal swab.*** Oropharyngeal swabs (Copan Transystem™ 140C rayon swabs in liquid Amies medium) will be collected from each participant by swabbing the posterior pharynx (both tonsils, posterior wall, uvula and any area of inflammation or exudation), avoiding touching the tongue, cheeks and lips. After sample collection, the swab be aseptically placed in liquid Amies transport solution and placed in a cold box until processing in the laboratory.

Oropharyngeal swabs will be collected in the same way for participants complaining of symptoms that could be consistent with acute pharyngitis at unscheduled visits

***Normal skin swab.*** Normal skin swabs (CITOSWAB ^®^ flocked nylon fibre mini-tip swabs in 1ml liquid Amies medium) will be collected with the intention of identifying any StrepA present on the skin, rather than differentiating skin site. Therefore, to maximise sensitivity, a single swab will be used on multiple skin sites.

Swabs will be obtained using modification of a standard skin microbiota swabbing technique ^28– 31^ in which the swab head is moistened with sterile water prior to skin swabbing. The swab will be taken from 2 by 2cm squares of skin on the forehead, then a larger area (5 by 20cm) on both forearms and both lower legs, and then placed aseptically in liquid Amies transport medium and stored in a cold box until processing in the laboratory.

SSS will be collected in the same was as NSS but from a 2 by 2cm patch of skin overlying typical non-infected scabies lesions.

***Pyoderma wound swab.*** Pyoderma WS (Copan Transystem) will be taken at from participants any visit with evidence of pyoderma (a skin infection with pus or crusts). Pus will be expressed if necessary. WS will be placed in liquid Amies transport medium in cold boxes until processing in the laboratory.

***Dried blood spot.*** DBS samples will be collected using dried blood spot collection cards (Whatman 903 protein saver snap-apart cards with four sample spots) from a finger prick on the participant. Four drops of blood will fill the four spots on the DBS card. The finger will be cleansed with alcohol and allowed to dry before the finger prick is made with a lancet. Following collection, the DBS card will be left to dry at room temperature before transportation. Transportation to the laboratory will be in a cold box.

***Blood sampling.*** The study team will be trained to perform venepuncture in the field. In the case that the head of the household, all participants aged over 18, and all guardians of children under 18 verbally consent to venepuncture for blood to be taken on site, this will be performed within the household. Alternatively, an appointment will be made at a specified time to attend Sukuta Health Centre where venepuncture for blood serum will be performed by members of the study team.

Peripheral blood will be collected into serum separation tubes using aseptic technique, ensuring appropriate PPE is used. BS samples will not be obtained from participants under the age of 2 years or those who do not verbally consent.

***Oral fluid samples.*** OF samples will be collected using an ORACOL ^®^ salivary collection device (Malvern Medical Developments, S10) from participants at the time points specified in Table 1. The oracol swab will be placed in the buccal cavity of the participant between the gums and the cheek for two minutes. Once obtained, the swab will be immediately placed in the collection tube according to the manufacturer’s instructions. OF tubes will be transported to the laboratory in a cold box.

***Environmental swabbing.*** At monthly household visits, two ES (Copan Transystem) will be taken from common touch points in the household. At the enrolment visit, the study team will identify two surfaces to swab within the household which are commonly touched by multiple people. Swabbing points might include door handles, table surfaces, curtains, benches, chair handles etc. Once two surfaces have been decided for the household, those will be the two surfaces swabbed at each subsequent visit.

The ES tip will be soaked in sterile water prior to swabbing and will be rubbed slowly and thoroughly over the surface (up to 50cm) three times reversing direction between strokes. Once collected, the swab will be aseptically placed in liquid Amies transport medium and stored in a cold box until processing in the laboratory.

For the personal and household hygiene visit, additional environmental swabs will be collected from a wider range of common touch points in the house, and a swab will be soaked in water taken from the household greywater source.

***Settle plates.*** A settle plate will be used at each monthly household visit to passively capture the presence of airborne StrepA within households. A culture petri dish pre-prepared with Colombia blood agar will be placed at a suitable point in the main social indoor room of the household. Ideal placement of the settle plate would be at least one metre off the floor, one metre away from the walls and other large obstacles. The plate will be left for one hour, then retrieved and stored in a cold box until processing in the laboratory.

### Laboratory evaluations

***Sample transport.*** All swabs and clinical samples taken in the field, except for DBS cards which will be dried first at room temperature, will be stored as soon as possible in a cold box maintained at approximately 2–8°C. All samples will be transported in the cold box to the MRCG Fajara laboratories for processing the same day.

***Culture procedures.*** OPS, NSS, SSS, WS and ES will be processed in the same way. Swabs will arrive at the laboratory in 1ml of liquid Amies transport medium. On arrival at the lab, after ensuring that the swab is inside and the lid is properly closed, the swab will be briefly vortexed in the transport medium. Next the swab will be removed and streaked onto a Colombia blood agar culture plate, and then discarded. Colombia blood agar was chosen for its availability MRCG and utility in culturing both *Streptococci* and *Staphylococci*, though use of selective blood agars may have been preferrable to prevent overgrowth. The remaining liquid Amies will be stored at -70°C without the addition of glycerol for subsequent use. It should be noted that the absence of glycerol in the stored medium precludes the possibility of reculturing from these samples, and the addition of glycerol will be considered in future studies.

The culture plates will be incubated for 18–24 hours at 37°C and assessed for the presence of beta-haemolytic colonies. Colonies with clear beta-haemolysis will be picked and replated for purity for a further 18–24 hours at 37°C. Pure growth colonies will then undergo catalase testing, and if negative then latex agglutination testing for Lancefield group A, C and G. Colonies identified from the primary plate as possible *Staphylococcus aureus* from NSS, SSS and WS will be identified based on their morphology and will also be replated for purity, then if catalase positive, will be tested for *S. aureus* using staphylococcal latex testing. Latex agglutination tests will be used for initial identification of streptococcal groups due to their practicality in our setting. However, we acknowledge the limitations of this method in distinguishing between *S. pyogenes* and SDSE. Liquid Amies transport medium from the swabs will be stored for future PCR validation, specifically targeting the *speB* gene.

Single colonies identified from the purity plate of any group A, C or G streptococci isolates identified will then be stored in glycerol broth at -70°C for later revival, DNA extraction and whole genome sequencing (WGS), which will provide detailed information on species, antigen presence and carbohydrate expression. *S. aureus* colonies will also be stored for later analysis. In future studies, consideration will be given to additionally storing a sweep of colonies from the original culture plate.

Antimicrobial susceptibility testing by disc diffusion using standard CLSI procedures will also be performed on group A, C or G streptococcal isolates identified.

***Whole genome sequencing (WGS).*** Isolates will be revived, and DNA extracted using established methods. Library preparations and WGS (Illumina short read and Oxford Nanopore technology long read platforms) will be undertaken. Quality control, *de novo* genome assembly, and core genome determination will be performed, followed by basic phylogenetic reconstruction using maximum likelihood. *Emm* and MLST typing and AMR will be performed. Genotypically-linked isolates will be determined by analysing genetic diversity and relationships between isolates.

***Dried blood spot processing.*** Upon arrival at the laboratory, DBS cards will be dried at room temperature overnight, then stored at -20°C for later elution. To elute the blood, 6mm punches of dried blood filter paper will be obtained and eluted using a buffer solution. The resulting eluate will then undergo serological analysis for anti-StrepA antibodies, including antibodies to Streptolysin O, SpyCEP, SpyAD, GAC, DNAseB, Enn, Mrp and M protein. Responses to the hypervariable M protein will be explored by selecting three types of antigens: first, *emm*-cluster-representative M peptides; second, M peptides from emm-types identified in The Gambia; and third, M peptides from *emm*-cluster-representative M protein vaccine antigens. A similar framework will be applied to selecting representative Mrp and Enn proteins. The antigens were chosen to provide a mixture of well-established markers of infection and to cover antigens used in some leading vaccine candidates. Our preliminary (unpublished) evidence suggests that DBS samples are adequate for monitoring the development of immunity to StrepA infections and carriage events compared to serum, as has been shown elsewhere ^32^. 

***Serum blood.*** BS samples will be used to assess serological activity to StrepA antigens including Streptolysin O, SpyCEP, SpyAD, GAC, DNAseB, Enn, Mrp, and M protein at baseline and at the end of the cohort. BS taken at the same time-points as DBS samples will additionally contribute to validation of DBS in this setting as a reliable and reproducible method for measuring anti-StrepA antibodies. Serum samples will be stored for further immunological work including streptococcal killing assays and opsonophagocytic assays to explore correlates of protection from StrepA asymptomatic carriage and clinical disease.

***Oral fluid samples*** OF samples will be mixed with antibody stabilising buffer on the day of collection. OF samples will be used to assess for mucosal antibody activity to StrepA antigens including Streptolysin O, SpyCEP, SpyAD, GAC, DNAseB, Enn, Mrp, and M protein. Samples will be stored for further immunological work.

### Modelling

Using data generated on swab positivity time, participant relationships, WGS data on phylogenetic relatedness of strains, geographic distance between households, and assortativity of social mixing in this setting, we will attempt to identify likely transmission events between individuals using R packages such as *outbreaker2* and *o2geosocial.* These models use Bayesian techniques to compute the likelihood that transmission occurred between individuals or not and hence allows for reconstruction of likely transmission chains.

Utilisation of the novel data in this project will allow estimation of relative contributions of between and within household transmission, and transmission between symptomatic and asymptomatic individuals. To our knowledge this has never been done for StrepA carriage and infection in Africa. The household model will also be valuable in evaluating potential intervention strategies for future implementation within LMICs. Once past events have been estimated, it will be possible to calibrate the model to simulate forward to predict likely onward transmission in the case of an individual with certain characteristics becoming positive.

### Sample size considerations

The primary outcome measures used to determine sample size were:

1. Monthly StrepA carriage prevalence, and
2. StrepA carriage and infection incidence over 12 months.

In HIC, StrepA pharyngeal carriage prevalence in children is 2–17% ^20, 21^, and in Uganda is 15.9% ^33^. Our study also includes adults, in whom carriage is lower, but will use pooled skin and pharyngeal carriage as our outcome measure, which will likely increase prevalence in turn. We therefore estimate a pooled prevalence of 15%.

StrepA pharyngeal carriage yearly incidence in children in the US was shown to be 27–32% ^20^. We found a skin infection incidence of 592/1000 child years in The Gambia during an influenza vaccine study follow-up (unpublished data) and of which ~50% are likely due to StrepA ^11^. As we are including adults with a likely lower incidence, we estimate a yearly incidence of 20%.

The sample size was calculated for the primary objective, StrepA carriage prevalence, using the formula below to measure the estimated prevalence of 15% with a precision of ±5%.


$$n=\frac{Z_{{\alpha /2}^{2}}\times p\times (1-p)}{e^{2}}$$

Where *p* is predicted proportion and *e* is desired precision.

Using *Z*=1.96 for *α*=0.05, *p*=0.15 and *e*=0.05 we require a sample of 196. Intracluster correlation is unknown, therefore we used a conservative design effect of 2, which allowing for 10% drop-out rate gives a required sample size of 431.

We therefore propose to recruit 45 households, which with an average household size of 10, will equal approximately 450 individuals for the main cohort.

This sample size would provide adequate power for precise estimates of prevalence and incidence of StrepA carriage (precision between ±4 and ±5%) and to detect risk factors for StrepA carriage with prevalence (or incidence) rate ratios of greater than 2 with 80% power.

### Data analysis

The clinical epidemiology of StrepA and SDSE carriage and infection will be presented using descriptive statistics. Baseline and monthly prevalence of skin and pharyngeal StrepA and SDSE carriage will be reported, including seasonal (monthly) variation. For pharyngeal and skin infection, baseline prevalence will be reported, then monthly and annual incidence for the duration of the study. The typical patterns of transmission observed between individuals within households will be described.

Logistic regression models will be used to look for socio-demographic and medical risk factors for carriage and infection at baseline. Survival analysis (extensions to Cox proportional hazards models) will be used to explore risk factors for carriage and infection throughout the study period taking into account household clustering, repeated events and time-dependent co-variates. The impact of carriage and infection in a close contact or family member in the month prior to new acquisitions of carriage or new infection will be investigated. The relationship between SDSE carriage and StrepA carriage and infection, whether SDSE presence impacts StrepA *emm*-type diversity, whether SDSE carriage is itself a personal risk factor for StrepA will also be explored. Additional risk factors to be explored include the impact of scabies, social mixing patterns, and socio-demographic factors.

To explore the protective association of antibody titres further, regression analysis will be performed to establish the association of antibody titre for each conserved antigen with incident disease, carriage or no carriage/infection accounting for covariates including age, sex and household size.

### Data collection and handling

Field data will be collected on electronic case report forms inputted on tablet computers by the field team. The questionnaires will be designed using REDCap ^TM^ electronic data capture software hosted at MRCG. Data will be collected offline and synced with the secure database at the end of each day. Data generated in the laboratory will be inputted onto the same database. Written informed consent will be sought from all participants prior to any study activities and before any data is collected.

Questionnaires will be designed with up-front data quality checks including reference ranges and dropdown menus to minimise incorrect data entry. Additionally, after completion of the study, a data checking process will be performed running queries to check for incomplete or nonsense data.

All data will be kept confidentially, and electronic data encrypted. Each participant will be assigned a unique study ID, so that no person identifiable data will be kept on the database. Any person identifiable data will be held securely and will not be available to anyone other than those in the investigator team. Data will be handled in accordance with the data management SOPs of MRCG which is fully compliant with GDPR regulations. Anonymised data will be held in the study database for a minimum of 10 years following project completion, in compliance with LSHTM’s Records Retention and Disposal Schedule. Anonymised raw study data and analysis code will be deposited in the LSHTM Data Compass repository on publication of study outputs and will be available upon request for scientific purposes.

Whole genome sequencing data will be generated using both short read and long read platforms. Raw sequence data will be in the form of fastq files and initially stored on high-performance clusters (HPCs). Data management and analysis will be performed on pipelines established on both the MRCG HPC and the University of Sheffield HPC, as well as cloud-based servers such as the MRC CLIMB platform. Raw sequence data will be archived at MRCG according to their data archiving procedures. Certain processed data fields from genomic analysis (e.g. *emm* type) will be included as variables in the study REDCap ^TM^ database. Sequence data along with links to relevant metadata will be submitted to a public sequence repository (e.g. genbank) as is standard practice, on publication of study outputs. Analysis pipeline and code will be made openly available via GitHub on publication of the study.

### Ethics and informed consent

This study will be conducted in accordance with the principles set forth in the ICH Harmonised Tripartite Guideline for Good Clinical Practice and the Declaration of Helsinki in its current version, whichever affords the greater protection to the participants.

Ethical approval has been obtained for the study from the MRC Scientific Coordinating Committee and the joint MRC/Gambia Government Research Ethics Committee, as well as the LSHTM ethics committee. Ethical approval reference number LEO24005.

Sensitizing potential study participants will precede the formal recruitment period to ensure that they are aware of the study as far in advance as is practical and therefore are given as much chance as possible to consider their potential involvement prior to providing informed consent. Sensitization will be approached using community and household/individual level strategies.

At the informed consent visit (at least 24 hours after sensitization), the study team will discuss the study with the household head and other household members to confirm that they have understood the consequences of study participation and to answer any remaining questions. If all the inclusion are met and none of the exclusion criteria are, the study team member will proceed to obtain informed consent from all household members.

To obtain informed consent from the household members, in the presence of a literate witness, a member of the study team will translate the informed consent document (ICD), which is in English, line-by-line into the local language spoken by the consenting individual (e.g. Mandinka or Wolof). Once the entire ICD has been translated, the study team member will answer any questions that the individual may have. If the consenting individual remain willing to participate and to provide informed consent, they will be asked to sign or thumbprint signature page of the ICD. If the participant is not literate, the witness will write the date and time and the participant will be asked to thumb-print the signature portion of the ICD. For participants under the age of 18 the child’s parent will be required to sign (or thumbprint) the ICD on their behalf. Children aged between 12 and 17 years inclusive will be asked to provide assent to participate in the study in addition to the informed consent provided by the child’s parent or legal guardian.

### Dissemination

This observational cohort study is registered on ClinicalTrials.gov (NCT05117528). The study results along with raw data and analysis code will be published promptly in peer-reviewed journals and promoted through the MRCG communications department and through social media where appropriate. Data will be submitted as abstracts to be presented at international conferences such as the European Congress of Clinical Microbiology and Infectious Diseases and the Lancefield International Meeting on Streptococci and Streptococcal Disease.

## Study status

Field work for this study is now complete. Study enrolment commenced on 27 ^th^ July 2021 and the final MV12 visit was completed on 28 ^th^ September 2022. 442 individuals were recruited from 44 households, with 3 households and 160 individuals being lost to follow up.

## Data Availability

No underlying data are associated with this article. Zenodo. REDCap data dictionary. DOI: https://doi.org/10.5281/zenodo.7463052 ^34^ Zenodo. SpyCATS informed consent documents (adult and child). DOI: https://doi.org/10.5281/zenodo.7501168 ^35^ Data are available under the terms of the Creative Commons Attribution 4.0 International license (CC-BY 4.0).
