# Supplementary material for: Streptococcus pyogenes carriage acquisition, persistence and transmission dynamics within households in The Gambia (SpyCATS): protocol for a longitudinal household cohort study
Source: Wellcome Open Res. 2023 Oct 30;8:41. Originally published 2023 Jan 27. [Version 2] doi: 10.12688/wellcomeopenres.18716.2 (PMC10638483; doi:10.12688/wellcomeopenres.18716.2)
Supplement: Peer review history

# Reviewer report: Reviewer response for version 2

**DOI:** 10.21956/wellcomeopenres.22555.r69402

**Published:** 2023-11-10

**Authors:** Stephan Brouwer

**Affiliations:** 1 The University of Queensland, Saint Lucia, Queensland, Australia

I would like to inform you that I am happy with the revisions, and I do not have any additional comments on the revised version.

Is the study design appropriate for the research question?

Yes

Is the rationale for, and objectives of, the study clearly described?

Yes

Are sufficient details of the methods provided to allow replication by others?

Partly

Are the datasets clearly presented in a useable and accessible format?

Yes

Reviewer Expertise:

NA

I confirm that I have read this submission and believe that I have an appropriate level of expertise to confirm that it is of an acceptable scientific standard.

# Reviewer report: Reviewer response for version 1

**DOI:** 10.21956/wellcomeopenres.20754.r64027

**Published:** 2023-08-24

**Authors:** Stephan Brouwer

**Affiliations:** 1 The University of Queensland, Saint Lucia, Queensland, Australia

**Summary:**

 This is a well-written and carefully designed study protocol by Armitage *et al*. that will provide valuable insights into the true burden of *Streptococcus pyogenes* disease in low-middle income settings. There is a limited understanding of evolution and transmission pathways in regions with the highest incidence *of S. pyogenes *diseases, such as Africa and the Pacific region. This longitudinal household observational study will make an important contribution to our understanding on the prevalence, incidence, and transmission dynamics of *S. pyogenes *in The Gambia. In addition, this study aims to investigate carriage and infection epidemiology of closely related *Streptococcus dysgalactiae *subsp. *equisimilis* (SDSE), which may also cause significant infections in humans and is highly prevalent in low-middle income settings. However, since the study aims to discriminate the two species and their specific contributions to health burden, I felt that there are a few limitations regarding the selection of molecular markers that may not allow to differentiate between *S. pyogenes *and SDSE (see below) with 100% confidence, thus rather reporting on the burden of “beta-haemolytic streptococci”.

**Major points:**

*S. pyogenes *and SDSE share extensive genetic homology, including genes encoding conserved virulence factors (e.g. *emm*, *slo*, *sls*, streptokinase and C5a peptidase). Moreover, several studies have reported the (rare) occurrence of Group A-positive SDSE, making it even more difficult to distinguish the two species. The authors suggest the use of latex agglutation tests for the identification of the streptococcal group. However, these have important limitations when trying to distinguish between *S. pyogenes *and SDSE (McMillan *et al.*, 2010 ^1^) and alternative or additional assays should be carried out to validate results, such as PCR amplification of the *speB *gene and the intergenic region upstream of the *scpG *gene (Davies *et al.*, 2007 ^2^, Watanabe *et al.*, 2016 ^3^, McMillan *et al.*, 2010 ^1^). Also, it may be very difficult to use antibodies to SLO, SpyCEP, SpyAD, GAC, DNAase B, Enn, Mrp and M protein as *S. pyogenes*-specific serological markers to explore *S. pyogenes *prevalence and infection rates given the genetic similarity to SDSE. How do the authors rule out the possibility that antibody titres are a correlate of SDSE infection?

 Page 11: ‘ *Dried blood spot processing*’: Antibodies to Enn, Mrp and M protein is very generic. There are more than 200 different *emm* types. It is not clear how the authors address this variability and possible issues with serotype specificity and/or (cross-)reactivity.

**Minor points:**

 Page 4: ‘ *To identify non-human reservoirs of StrepA within households’*. The term “reservoir” is misleading and incorrect as it implies that *S. pyogenes* flourishes and multiplies or can be permanently maintained in these environments, which is not the case. Please change.

 Page 5: ‘ *Exclusion criteria*’: Can the authors please provide an example how team members judge a participant unable or unlikely to participate and comply?

 Page 5: In ‘ *Field activities*’ it is stated that enrolments have commenced in July 2021. Are enrolments currently still ongoing, or are they finalised? Can the authors please provide an update on the enrolment situation and also comment on the participation success rate? Also, I understand that enrolment commenced at a time when the country witnessed a severe wave of COVID-19 infections that was mainly associated with the delta variant from July to September 2021 (Gambia-COVID-19-Situational–Report_2021_7th_8th_November_No-399). Have participants with or without symptoms been tested for COVID-19? These would be important data to include in this study. The authors should also comment on the possible impact this wave may have had on carriage/infection rates. Could these data be used to investigate a potential correlation of co-infection?

Is the study design appropriate for the research question?

Yes

Is the rationale for, and objectives of, the study clearly described?

Yes

Are sufficient details of the methods provided to allow replication by others?

Partly

Are the datasets clearly presented in a useable and accessible format?

Yes

Reviewer Expertise:

Bacterial pathogenesis, Streptococcus pyogenes, host-pathogen interaction

I confirm that I have read this submission and believe that I have an appropriate level of expertise to confirm that it is of an acceptable scientific standard, however I have significant reservations, as outlined above.

## References

[1] : Molecular markers for discriminating Streptococcus pyogenes and S. dysgalactiae subspecies equisimilis. *Eur J Clin Microbiol Infect Dis* .2010;29(5) : 10.1007/s10096-010-0899-x 585-9 10.1007/s10096-010-0899-x 20221892

[2] : Virulence profiling of Streptococcus dysgalactiae subspecies equisimilis isolated from infected humans reveals 2 distinct genetic lineages that do not segregate with their phenotypes or propensity to cause diseases. *Clin Infect Dis* .2007;44(11) : 10.1086/516780 1442-54 10.1086/516780 17479940

[3] : Severe invasive streptococcal infection by Streptococcus pyogenes and Streptococcus dysgalactiae subsp. equisimilis. *Microbiol Immunol* .2016;60(1) : 10.1111/1348-0421.12334 1-9 10.1111/1348-0421.12334 26762200

# Reviewer report: Reviewer response for version 1

**DOI:** 10.21956/wellcomeopenres.20754.r64025

**Published:** 2023-08-18

**Authors:** Shiranee Sriskandan

**Affiliations:** 1 Gram-Positive Molecular Pathogenesis Group, Department of Infectious Diseases, Imperial College London, Hammersmith Hospitals campus, London, W12 0NN, UK

The protocol for the household study of Streptococcus pyogenes acquisition and transmission dynamics in The Gambia is adequately detailed and easy to follow. Of note the fieldwork for this study is complete; comments are made with that knowledge. The primary aims of the study are likely to be met, provided that adequate numbers of children are recruited in the households, and the predicted asymptomatic carriage of 15% is broadly correct. A few clarifications in the protocol will enable others to follow or repeat what was done.

**1. Bacteriology:**

- 1.1: Environmental samples. Columbia Blood Agar has been used; as a suggestion, selective blood agar might be helpful to avoid overgrowth.
- 1.2: Human swabs. Copain Liquid Amies medium swabs are used - these are equivalent (if not better) than gel transport medium for recovery of bacteria. In this study, liquid medium is used after vortexing (Van Horn *et al.*, 2008 ^1^). The protocol states the tubes are vortexed and the swabs are used for plating - please clarify.
- 1.3: The liquid Amies medium is being stored frozen at -70; is this with or without glycerol? 
- 1.4: Groups A, C, or G streptococci are being stored in glycerol for WGS; are single colonies or a sweep of colonies from the original plate stored.
- 1.5: How is probable Staphylococcus aureus identified? (prior to catalase testing).

**2. Serological responses:**

- 2.1: Serum at time zero and at 12 months is collected with blood spots in between. Given that the study will capture incident active S. pyogenes infections and carriage events, is there preliminary evidence that the blood spots are adequate to monitor development of immunity to the infection?
- 2.2: Eight antigens are proposed for measurement of immunity; is there any explanation of how/why these were selected? (the authors have recently published data using an overlapping slightly different panel of antigens).
- 2.3: Is there any control measurement to be made in the samples, for example total IgG or a vaccine-related antibody response that is expected to be uniform in the population?

**3. Sample size and the model:**

- 3.1: Recently published cross sectional studies from Yemen and Ethiopia suggest asymptomatic carriage rates of 12-15% in children; these data support a predicted rate of 15% and the planned sample size however these are based on children only. How many children are expected in study?
- 3.2: The protocol states that household size is expected to be 10/household, but also says the 2013 data yielded a figure of 8 per household. Which is correct (the latter might yield only 360 participants)?
- 3.3: In terms of transmission, is it an aim to determine the effect of strain type on transmission (for example comparing two types of strain or two types of carriage)?

**4. Subgroup for frequent swabbing:**

 The protocol states 16 households will be selected for intense swabbing, but then states that, in the second half of the study, 4 households will undergo intense swabbing. Does this mean that in fact 4 households (out of a possible 16) will eventually undergo this process - or that 16 households will undergo intense swabbing in the first half and four of these will repeat this in the second half? 

**5. Information about social mixing:** 

 The focus of the protocol is the household which - if understood correctly - may include multiple smaller outbuildings. If so, please clarify if information about shared sleeping vs cooking/eating arrangements are collected. Although information about non household contacts is being collected, is place and name of school or work collected, since valuable information might be obtained therein?

**6. Ethics:**

 The study has been approved locally and at the sponsor institution. I was surprised that assent was not required from children under the age of 12 but assume this is acceptable in the region being studied. There is no comment about pregnant women and babies but one assumes they will be part of the study.

Is the study design appropriate for the research question?

Yes

Is the rationale for, and objectives of, the study clearly described?

Yes

Are sufficient details of the methods provided to allow replication by others?

Partly

Are the datasets clearly presented in a useable and accessible format?

Not applicable

Reviewer Expertise:

Streptococcal clinical research: transmission and pathogenesis

I confirm that I have read this submission and believe that I have an appropriate level of expertise to confirm that it is of an acceptable scientific standard.

## References

[1] : Comparison of the Copan ESwab system with two Amies agar swab transport systems for maintenance of microorganism viability. *J Clin Microbiol* .2008;46(5) : 10.1128/JCM.02047-07 1655-8 10.1128/JCM.02047-07 18353935PMC2395074
